# Ameliorative Effects of *Spirulina platensis* Protein Hydrolysate on Oxidative Stress and Dyslipidemia in Model Animal

**DOI:** 10.3390/foods15132399

**Published:** 2026-07-06

**Authors:** Ahmad Ali, Sanaullah Iqbal, Azmatullah Khan, Imtiaz Rabbani

**Affiliations:** 1 Department of Food Science and Human Nutrition, University of Veterinary & Animal Sciences, Lahore 54000, Pakistan; ahmad.ali@uvas.edu.pk (A.A.); azmat.khan@uvas.edu.pk (A.K.); 2 Department of Physiology, University of Veterinary & Animal Sciences, Lahore 54000, Pakistan; imtiaz.rabbani@uvas.edu.pk

**Keywords:** *Spirulina platensis* protein hydrolysates, *Spirulina platensis* protein extract, hyperlipidemia, oxidative stress, animal model

## Abstract

Spirulina-derived protein hydrolysates (SPPHs) have attracted considerable attention as bioactive agents due to their potential metabolic and physiological benefits. This study evaluated the therapeutic efficacy of different enzyme-specific SPPHs—Pepsin (SPPH-P), Trypsin (SPPH-T), Chymotrypsin (SPPH-C), and a combined hydrolysate (SPPH-PTC)—in high-fat diet (HFD)-induced male Wister rats, compared with *Spirulina platensis* protein extract (SPPE, formulated using freeze–thaw cycles and ultrasonication followed by centrifugation) and atorvastatin as a Positive Control. The animals were randomly allocated into seven groups (n = 6 per group) and received their respective treatments orally for 4 weeks. Across treatment groups, significant improvements in obesity-related anthropometric indices were observed, including reductions in BMI, Lee Index, and abdominal circumference to thoracic circumference ratio (AC:TC), with the strongest effects noted in the atorvastatin and SPPH-PTC groups. Protein metabolism markers showed enhanced hepatic and serum protein status, reflected by increased albumin and total protein concentrations. Lipid profile analysis revealed marked decreases in total cholesterol, triglycerides, and LDL in both serum and liver homogenates, while HDL exhibited non-significant but favorable elevations. Liver function markers (bilirubin, ALT, AST) and renal parameters (uric acid, BUN) demonstrated notable improvements, particularly in enzyme-derived hydrolysate groups and Positive Control. Antioxidant assessments indicated substantial reductions in MDA levels and significant increases in SOD, CAT, and GSH activities in serum and liver tissues, confirming enhanced oxidative stress resistance. Among all treatments, SPPH-PTC consistently produced the most robust therapeutic outcomes. Overall, Spirulina protein hydrolysates, especially the combined PTC formulation, exert comprehensive beneficial effects on metabolic regulation, hepatic and renal function, and oxidative balance. These findings support their potential application as functional bioactive agents for managing obesity-associated metabolic disturbances.

## 1. Introduction

The alteration in lipid profile parameters resulting from disrupted lipid metabolism is termed as dyslipidemia [1]. Dyslipidemia is also considered a major risk factor for multiple non-communicable ailments, including metabolic syndrome and cardiovascular diseases [2]. Due to mega-production in food factories and the shift in masses towards Western diets (rich in saturated fats and calories), along with the sedentary lifestyle, the incidence of hyperlipidemia is already at an alarmingly high rate [3,4]. The oxidative modification of lipoproteins can occur secondary to elevated circulating lipids in the blood vessels, which not only contributes to atherosclerosis but can also amplify oxidative stress in the vascular compartment [5]. The oxidative stress originating from lipid peroxidation can be determined by malondialdehyde (MDA); whereas, the antioxidant potential is evaluated using catalase (CAT), superoxide dismutase (SOD) and glutathione (GSH). Increased level of circulating lipids often amplifies production of reactive oxygen species (ROS), which in turns exacerbates lipid peroxidation and aggravates dyslipidemia [6,7]. Moreover, multiple animal studies have documented the organ dysfunction and generation of oxidants in the heart and liver tissues after hyperlipidemia [8].

Although the allopathic countermeasures of hyperlipidemia are effective and easily available, their long-term use can lead to the development of adverse side effects, including gastrointestinal upset, micronutrient deficiencies (B-12), hepatotoxicity, and myopathies [9]. This has led to an increasing focus on identifying bioactive compounds in foods that can effectively modulate lipid metabolism and reduce hyperlipidemia [10]. Among various food-derived bioactive sources, microalgae, particularly *Spirulina platensis*, have attracted significant attention due to their rich nutritional and bioactive profile [11].

*Spirulina platensis* is a filamentous blue-green algae with an abundance of proteins (up to 70%), which has gained considerable interest from the scientific community as it possesses a nutritionally balanced composition, high protein quality indices comparable to meat, essential fatty acids (omega-3 and 6), and the presence of phytonutrients, i.e., phycocyanin and β-carotene [12]. Several studies have reported the beneficial effects of raw Spirulina on lipid metabolism, glycemic control, oxidative stress, and inflammation [13,14]. However, the bioavailability and biological activity of intact Spirulina proteins are limited due to their macromolecular nature. In this context, enzymatic hydrolysis can be crucial in releasing short-chain bioactive peptides, which exhibit enhanced antioxidant, antihypertensive, and hypolipidemic activities [15].

Spirulina protein hydrolysates (pepsin-based) were found to be more effective against whole Spirulina proteins in a high-fat-diet-fed mice model. The administration of hydrolysates derived from Spirulina led to a decrease in weight (40%), glucose (24%) and lipid profile markers (21% reduction in cholesterol). These effects were further validated by gene expression involving upregulation of the markers of lipid metabolism including AMPK, PPAR and adipocytokines indicating the potential role of Spirulina hydrolysates in managing obesity and related complications [16]. Similar findings were also observed in a Protamex-based Spirulina hydrolysates [17]. Protamex is a microbial endopeptidase and SPPH made with this microbial enzyme showed hypolipidemic effects by downregulating lipogenic genes (SREBP-1c, ACC, PPARγ) and upregulating AMPK and PPARα in the liver [17].

In another study, the alkaline proteinase and Protamex-based SPPH were observed with antifatigue performance in mice by enhancing antioxidant enzyme activity and reducing oxidative stress and fatigue markers. These effects were due to modulation of AMPK signaling and promoting lipid utilization, which also indicates the potential role of Spirulina hydrolysates in lipid metabolism [18]. Collectively, these findings indicate that Spirulina protein hydrolysates exert multiple physiological benefits through modulation of lipid metabolism and oxidative stress pathways.

Although these beneficial effects have also been reported for whole Spirulina preparations, the available evidence differs considerably from studies using protein hydrolysates. These hypolipidemic and anti-inflammatory effects were also observed in the current literature but it was mainly limited to whole Spirulina powder [19] or the Spirulina aqueous extract [20].

The enhanced biological activity of protein hydrolysates is thought to result from the release of bioactive peptides during controlled proteolysis. Controlled proteolytic hydrolysis often releases the bound bioactive peptides, smaller in size, possessing enhanced physiological abilities [21]. These peptides can ameliorate hyperlipidemia via interacting with enzymes responsible for lipid regulation, signaling pathways, and modulation of lipid and cholesterol synthesis, and lipoprotein metabolism [22].

In our previous study, Spirulina whole protein was enzymatically hydrolyzed using pepsin, trypsin and chymotrypsin. Consequently, these hydrolysates were characterized based on antioxidant potential, nutritional profiling and bioactive compounds. These hydrolysates exhibited enhanced antioxidant activity and consisted of low molecular weight peptides (<10 kDa) with maximum retention of nutritional quality, compared to raw Spirulina protein [23]. These findings provided the basis for assessment of in vivo antioxidant and hypolipidemic potential of these Spirulina hydrolysates.

Despite these promising findings, several limitations remain in the current literature. Most previous studies investigating the lipid-lowering effects of Spirulina hydrolysates have been limited to either in vitro models or in vivo experiments employing a single proteolytic enzyme. In contrast, the present study utilized a combination of gastrointestinal enzymes—pepsin, trypsin, and chymotrypsin—to better simulate human digestive conditions. Furthermore, we evaluated not only individual enzyme-derived hydrolysates but also their combined hydrolysate profile, providing a more physiologically relevant and comprehensive assessment. To the best of our knowledge, this multi-enzyme and combined hydrolysate approach offers a novel perspective in understanding the metabolic effects of Spirulina-derived peptides. Therefore, the present study was designed to evaluate the hypolipidemic and antioxidant potential of Spirulina protein-derived hydrolysates prepared from pepsin, trypsin, and chymotrypsin in a high-fat-diet-fed rat model.

## 2. Materials and Methods

### 2.1. Chemicals and Reagents

All the chemicals and reagents used in this experiment were of analytical grade and were purchased from Sigma Aldrich (Darmstadt, Germany), unless otherwise stated. The biochemical diagnostic kits were purchased from Bioactive (Santa Clara, CA, USA) for the quantification of serum proteins (total proteins; cat no. 104989993190, albumin; cat no. 104989993188), liver enzymes (ALT; cat no. 104989993175, AST; cat no. 104989993177, AP, Total bilirubin; cat no. BL010500050, Direct bilirubin; cat no. BL021000200, and Indirect bilirubin; cat no. BL022500500), lipid profile (HDL; cat no. HC010500050, LDL; cat no. 1279910329, Triglycerides; cat no. 1279910330, and total cholesterol; cat no. 104989993203), and renal function tests (creatinine; cat no. 104989993186, urea; cat no. 104989993202, uric acid; cat no. 1279910301, blood urea nitrogen; cat no. 104989993300). The animal feed (basal-10% fat and high-fat diet-60% fat) and the *Spirulina platensis* whole powder were procured from Scientific Traders Pakistan Private Limited (Lahore, Pakistan).

### 2.2. Animal Procurement and Housing

Healthy male Wistar rats (n = 42, age 8 weeks) with an initial weight of 200 g were purchased from the animal breeding facility of the University of Veterinary and Animal Sciences, Lahore. The rats were provided with an *ad libitum* basal diet and were given 1 week for acclimatization. All the rats were housed in controlled physical conditions with temperature (25 ± 2 °C) under 12 h light and dark cycles.

### 2.3. Formation of Spirulina platensis Protein Hydrolysates (SPPH) and Spirulina platensis Protein Extract (SPPE)

SPPE was obtained utilizing 5% Spirulina aqueous solution, followed by repeated freeze–thaw cycles (freezing at −20 °C and thawing at room temperature), ultrasonication (450 W for 30 min, pulse mode: 9 s on and 6 s off) and centrifugation at 8050 RPM for 45 min. Three proteolytic enzymes were used in the formation of SPPH which were pepsin, trypsin, and chymotrypsin. For pepsin, trypsin, and chymotrypsin, 4500, 2000, and 1000 IU/mL enzyme concentrations were used for hydrolysis. The hydrolysis conditions for pepsin were kept at pH 2 and 37 °C for 2 h. For trypsin and chymotrypsin, pH and temperature were kept at 8 and 42 °C, respectively, for 3 h. The hydrolysis condition for pepsin was adopted from [24]. Hydrolysis conditions from [25] were followed for trypsin and chymotrypsin. Owing to the unique ligation sites (pepsin ligates N-terminal of aromatic amino acids, trypsin ligates C-terminal of basic amino acids and chymotrypsin ligates C-terminal of aromatic amino acids) and to simulate protein digestion in humans, pepsin trypsin and chymotrypsin were used to make Spirulina hydrolysates. Moreover, it also increases the uniqueness and diversity of peptides obtained in hydrolysates, increasing their potential effectiveness [26]. The characterization of these hydrolysates has been described previously [23].

### 2.4. Induction of Hyperlipidemia via a High-Fat Diet

All the rats were placed on a lard-based high-fat diet (HFD-D12492) mainly providing 60% calories from fats for six weeks [27]. Afterwards, the lipid profiles were assessed to confirm the presence of hyperlipidemia. The total cholesterol, triglycerides, and LDL levels of more than 150, 150, and 120 mg/dL, respectively, indicated the induction of hyperlipidemia in rats.

### 2.5. Experimental Design

After the induction of hyperlipidemia, rats were randomly divided into 7 groups (n = 6 per group) using R software Version 4.6.1. The Negative Control group consisted of HFD fed rats that received no treatment, while the Positive Control group was administered with atorvastatin (40 mg/kg). The *Spirulina platensis* protein extract (SPPE) group received SPPE at a dose of 150 mg/kg. Similarly, the groups treated with *Spirulina platensis* protein hydrolysates prepared using different enzymes—pepsin (SPPH-P), trypsin (SPPH-T), and chymotrypsin (SPPH-C)—were each administered 150 mg/kg of their respective hydrolysates. In addition, a combined enzymatic hydrolysates group was given a mixture of pepsin-, trypsin-, and chymotrypsin-derived hydrolysates, which were prepared separately but mixed (1:1:1, *w*/*w*, in equal proportions) and given at the same dosage of 150 mg/kg.

### 2.6. Blood Collection Liver Homogenate Preparation

After 8 weeks, all the rats were euthanized, and blood sampling was performed using the heart puncture technique, followed by biochemical analysis after serum separation using centrifugation (3000 rpm, 10 min). Liver tissues (0.1 g in 0.9 mL PBS) were taken and washed using phosphate-buffered saline and were immediately stored in −80 °C for further analysis. For homogenate preparation, liver samples were centrifuged at 8000 g for 15 min at 4 °C after mechanical homogenization in an ice-cold phosphate buffer (pH 7.4) at a 1:9 (*w*/*v*). Serum parameters are expressed in standard units, i.e., mg/dL and U/L; whereas, liver homogenate parameters were expressed as mg/mL and U/mL following standard biochemical assay protocols.

### 2.7. Biochemical Analysis

The lipid profile (TGs, LDL, HDL, total cholesterol), liver enzymes (ALT, AST, AP, total bilirubin, direct, and indirect bilirubin), serum proteins (total proteins and albumin), and renal function tests were performed for both serum and liver homogenate samples using Bioactive diagnostic kits (Bioactive, Santa Clara, CA, USA). The serum and liver homogenate antioxidant and lipid peroxidation analysis was performed for superoxide dismutase, catalase, glutathione reductase, and MDA.

#### 2.7.1. Malondialdehyde; Thiobarbituric Acid-Reactive Substances (TBARS) Assay

Lipid peroxidation assay was performed using TBARS, previously documented in [28]. Separate reaction tubes were prepared for serum and liver homogenate samples, along with a blank after mixing 0.5 mL sample/blank, 0.5 mL phosphate buffer (pH 7.4), 0.1 M freshly prepared ascorbic acid, and 0.1 M ferric chloride. After thorough mixing, incubation was performed at 37 °C (1 h) for lipid peroxidation. Reaction was stopped with the addition of 1 mL TCA (10%), and centrifugation was performed at 3000 RPM (10 min). Afterwards, 1 mL supernatant was added with 0.67% TBA (1 mL) followed by mixing. Heating was done at 95 °C, after which ice cooling was performed. Absorption was taken at 532 nm using a UV-Visible spectrophotometer. Lipid peroxidation was calculated using the equation below.

MDA (µM/L): (Abs Sample − Abs Blank) × 6.41

#### 2.7.2. Catalase

Briefly, 100 μL of serum/ liver homogenate was mixed with 1 mL of 20 mM hydrogen peroxide (H_2_O_2_) prepared in 50 mM sodium–potassium phosphate buffer (pH 7.4) and incubated at 37 °C for 3 min. The reaction was stopped by adding 4 mL of 32.4 mM ammonium molybdate solution, and the absorbance was measured at 374 nm against a blank. Appropriate controls and standards were included in parallel. Catalase activity was calculated using the equation below, following the instructions of [29].

Catalase Activity (kU/L) = (2.303/t) × log (S_1_/S − M)) × (Vt/Vs)

where t is the reaction time (3 min), S is the absorbance of the test, M is the absorbance of the control-test, $S_{1}$ is the absorbance of the standard, $V_{t}$ is the total reaction volume (5100 μL), and $V_{s}$ is the sample volume (100 μL). All reagents were prepared fresh or stored appropriately, and measurements were performed in triplicate to ensure accuracy.

#### 2.7.3. Superoxide Dismutase

Superoxide dismutase (SOD) activity in serum and liver homogenate was determined using a colorimetric assay based on the inhibition of Nitro Blue Tetrazolium (NBT) reduction by superoxide radicals generated through the NADH–Phenazine Methosulfate (PMS) system [30]. Briefly, the reaction mixture contained 1.2 mL of 0.05 M sodium pyrophosphate buffer (pH 8.3), 0.5 mL of 50 mM phosphate buffer (pH 7.4), 0.1 mL of 1 mg/mL NBT, 0.1 mL of 0.1 mM NADH, 0.1 mL of 0.1 mM PMS, 0.5 mL of distilled water, and 0.5 mL of serum enzyme extract. For the control, the enzyme extract was replaced with phosphate buffer. The reaction was initiated by adding PMS and incubated at room temperature (25–30 °C) for 5 min in dim light. The reaction was stopped by adding 3 mL of n-butanol: glacial acetic acid (96:4 *v*/*v*), followed by vigorous shaking for 30 s to extract the blue formazan into the organic phase. After phase separation, the absorbance of the upper layer was measured at 560 nm. SOD activity was expressed as units per mL, where one unit is defined as the amount of enzyme required to inhibit 50% of NBT reduction under assay conditions, and percent inhibition was calculated using the equation below

% inhibition = [(A control − A sample) ÷ A control} × 100]

#### 2.7.4. Glutathione Reductase

Glutathione (GSH) levels in serum were estimated using the colorimetric method of [31] based on the reaction of free–SH groups with 5,5′-dithiobis (2-ni trobenzoic acid) (DTNB). Briefly, 50 μL of serum was diluted with 250 μL of 10 mM phosphate buffer (pH 7.4) and 100 μL of 0.15 M Tris-HCl buffer (pH 8.0), followed by centrifugation at 10,000 rpm for 10 min. For the assay, 0.1 mL of the supernatant was mixed with 2.4 mL of 0.02 M EDTA and incubated on ice for 10 min. After adding 2 mL of distilled water and 0.5 mL of 50% trichloroacetic acid (TCA), the mixture was incubated again on ice for 10–15 min and centrifuged at 3000–3500 rpm for 10 min. One mL of the clear supernatant was then combined with 2 mL of 0.15 M Tris-HCl and 50 μL of freshly prepared 6 mM DTNB, vortexed, and allowed to react for 2–3 min. The yellow-colored product (TNB) formed was measured at 412 nm using a spectrophotometer. GSH concentrations in samples were calculated from a standard curve prepared using known concentrations of reduced glutathione, with all reagents freshly prepared and blanks included for calibration. Glutathione (GSH) used in this study refers to the reduced form of glutathione, a well-known endogenous tripeptide antioxidant and metabolite, rather than an enzyme.

### 2.8. Liver Histopathology

The formalin-preserved organs were subjected to histopathological analysis following the instructions of [32].

### 2.9. Statistical Analysis

Data were analyzed using R statistical software version 4.5.1 and expressed as mean ± SD and percentages. Data normality was assessed through the Shapiro–Wilk and Kolmogorov–Smirnov tests with *p* value > 0.05. considered normally distributed. The differences among biochemical parameters of the serum and the liver homogenate were tested using one-way ANOVA. The intergroup differences were measured using the Duncan Multiple Range Test (DMRt). The level of significance was kept at 5%.

## 3. Results and Discussion

**Figure 1:**
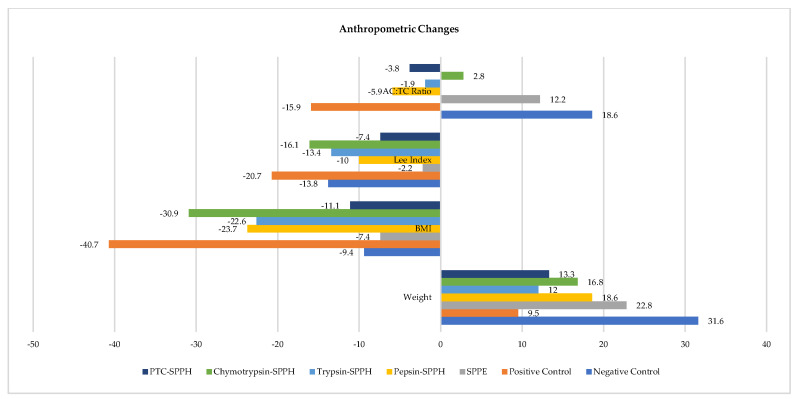
Change in anthropometric parameters of HFD-induced hyperlipidemic rat model after 2 months of SPPH administration.

All the treatment groups showed significant weight loss, with trypsin-SPPH showing a marked difference (31.52–12.04%) while the least difference was observed in the Positive Control group (31.52–9.46%) as compared to the Negative Control. Across all treatment groups, both BMI and Lee Index showed a significant reduction from baseline over two months. The greatest decline in BMI was observed in the Positive Control group (40.89%) as compared to the Negative Control (7.28%), followed by chymotrypsin-SPPH (29.4%). A similar trend was observed for Lee Index, with the highest reduction in the Positive Control group (20.44%) as compared to the Negative Control (2.27%), followed by chymotrypsin-SPPH (15.44%). Although all the treatment groups showed an increase in abdominal circumference, the highest increment was observed for the Negative Control (16.42%), followed by the treatment group SPEE (12.88%). However, the least increase was noted in the Positive Control group (2.29%). Compared to the negative control (19.98%), the AC: TC ratio depicted a significant reduction in all study groups, with the highest decline in the Positive Control group (15.84%), except SPPE and chymotrypsin-SPPH, showing an increase in the AC: TC ratio (12.62% and 3.82% respectively) (Figure 1). Beyond improvements in anthropometric parameters, the intervention also influenced protein metabolism and hepatic nutritional status, as reflected by changes in albumin and total protein concentrations.

Since hepatic protein synthesis is closely linked with systemic protein homeostasis, serum protein biomarkers were further evaluated to determine whether these localized hepatic effects were translated into circulating biochemical changes. Regarding albumin concentration in liver homogenates, a statistically significant difference was observed across all study groups (*p* < 0.05). The most pronounced increase in albumin level was shown by the Positive Control group (129.16%), while the least increase was shown by the treatment group, pepsin-SPPH (40.77%), as compared to the Negative Control. For total protein concentration in liver homogenate, although all study groups showed increments, no statistically significant difference was observed (*p* > 0.05).

In terms of serum albumin and total protein concentration, all the study groups showed a statistically significant increase as compared to the Negative Control (*p* < 0.05). The maximum increase in serum albumin was observed in the Positive Control (29.66%), followed by the treatment group PTC-SPPH (16.31%), as compared to the Negative Control (2.21 mg/mL). For serum total protein concentration, the treatment group chymotrypsin-SPPH showed a remarkable increase (22.65%); however, the difference across the study groups was statistically insignificant *p* > 0.05.

Following the observed improvements in protein-related biomarkers, lipid metabolism was investigated to assess whether Spirulina protein hydrolysates could also modulate dyslipidemia associated with metabolic stress. Regarding liver homogenate, the *p*-value < 0.05 shows a statistically significant reduction in total cholesterol, triglycerides, and LDL concentration among all study groups. In terms of total cholesterol, the treatment group PTC-SPPH showed a remarkable decline (22.01%), followed closely by the Positive Control (20.59%), as compared to the Negative Control. For triglyceride concentration, trypsin-SPHH showed a pronounced decrease (24.35%) followed by SPEE (18.24%), as compared to the Negative Control. As per LDL concentration, a similar trend was observed, with PTC-SPPH showing the highest reduction (38.56%), followed by Positive Control (37.11%) in comparison to the Negative Control. Although the difference was statistically insignificant, an exceptional trend was observed for HDL concentration across all study groups, with Positive Control and PTC-SPPH showing an increase, while other treatment groups depicted a decrease in HDL levels. Regarding serum total cholesterol, triglycerides, and LDL concentration across all study groups, there was a statistically significant reduction (*p* < 0.05). In terms of total cholesterol, a trend like liver homogenate was observed. The treatment group PTC-SPPH showed the highest reduction (12.28%) followed closely by the Positive Control (11.17%), as compared to the normal control. However, the treatment group SPEE showed the least decline (2.65%) (Table 1).

**Figure 2:**
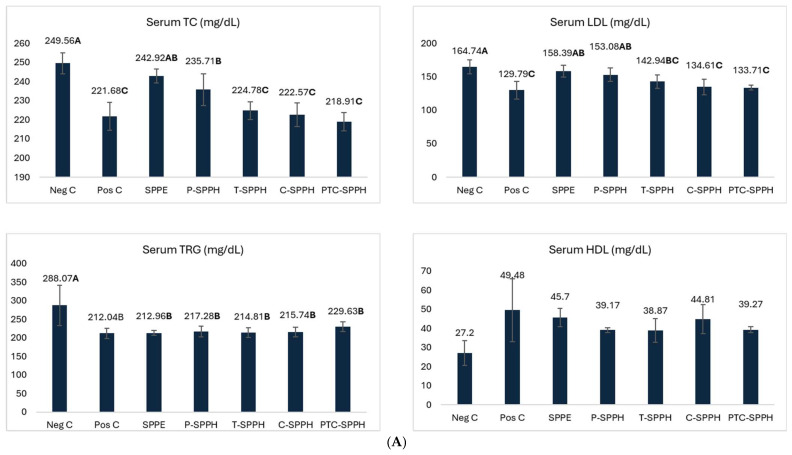

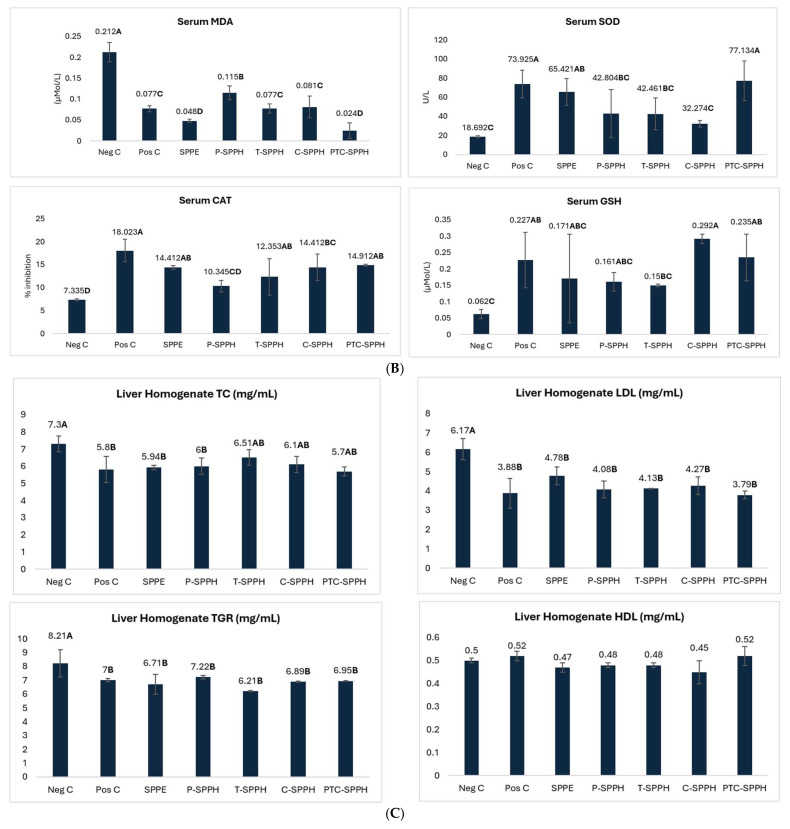

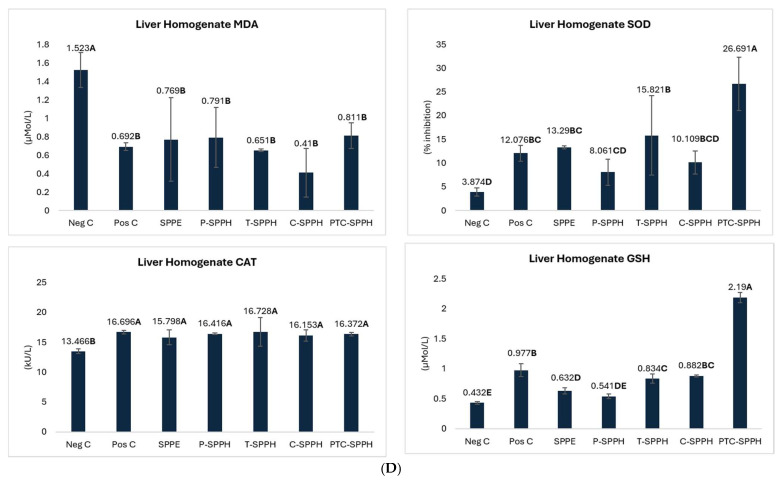
Effect of SPPH on lipid profile and antioxidants in HFD-induced hyperlipidemic rat model (Serum/Liver Homogenate). (**A**) Lipid profile in serum samples; (**B**) serum antioxidant enzymes and metabilites; (**C**) liver homogenate lipid profile; (**D**) liver homogenate antioxidant enzymes and lipid profile. Bold letters in figures represent significant different groups.

As per serum triglyceride levels, the reduction was comparable among all the study groups, with the Positive Control group and SPPE showing the highest reduction (26.39% and 26.07%, respectively) and PTC-SPPH showing the least decrease (20.28%) as compared to the Negative Control. For LDL concentration, the Positive Control depicted the highest reduction (21.21%), while the minimum reduction was recorded for the treatment group SPPE (3.85%) in comparison to the Negative Control. Although all the study groups showed an overall increase in Serum HDL levels, the difference in the level of HDL was statistically insignificant (*p* > 0.05). (Figure 2). The lipid-lowering effects observed across treatment groups suggest that the bioactive peptides generated through enzymatic hydrolysis may differentially regulate lipid metabolism depending on enzyme specificity and peptide composition.

Spirulina protein hydrolysates have been explored for their lipid lowering ability in various studies. As evident by the outcomes of the present study, administration of Spirulina protein hydrolysates improved the lipid profile by decreasing serum as well as liver TC, TG, and LDL-C and slightly increasing HDL-C. Some treatment groups showed more pronounced improvement in lipid profile as compared to others owing to uniqueness of ligation sites of these enzymes for hydrolysis. For instance, pepsin mainly breaks aromatic amino acids containing peptide bonds under acidic conditions, resulting in peptides with greater hypolipemic effect. In contrast, trypsin targets peptide bonds from the carboxyl side of arginine and lysine residues, producing distinct structural peptides that may affect lipid metabolism pathways [33]. Similarly, Chymotrypsin preferentially cleaves aromatic amino acids such as phenylalanine, tyrosine, and tryptophan, generating peptides having varied hydrophobicity and biological functionality. Similar patterns have been observed in previous studies, where antihyperlipidemic potential of bioactive peptides, obtained from algal proteins were significantly influenced by enzymatic specificity [34]. These variations are also demonstrated in a similar study on quinoa protein hydrolysates [35]. These differential responses among hydrolysates may further be explained by the enzyme-dependent generation of structurally distinct peptides with varying biological activities.

In addition to general hypolipidemic mechanisms, enzyme-specific cleavage patterns may further determine the magnitude and nature of these metabolic effects. Thus, these findings align with recent research indicating reduced blood cholesterol and improved lipid metabolism by Spirulina hydrolysate supplementation [36]. This hypolipidemic effect may be attributed to the pancreatic lipase lowering ability of bioactive peptides via various hydrophobic and hydrophilic interactions, thereby reducing lipid synthesis and increasing lipolysis. Importantly, this decline in TC and lipoproteins suggests improved lipid metabolism and utilization either due to decreased cholesterol synthesis or increased lipolysis. These results are aligned with the study demonstrating that the bioactive peptides contribute to increase in HDL-C, thereby ensuring cardioprotective potential [37].

Collectively, these findings emphasize that the biological functionality of Spirulina-derived peptides is strongly influenced by enzymatic hydrolysis conditions and peptide characteristics. Owing to the specific cleavage site, trypsin generates smaller, positively charged peptides with increased biological activity. Studies have reported that greater functional properties have been demonstrated by trypsin-derived Spirulina hydrolysates compared with other enzymatic protocols, likely due to the presence of basic amino acids in bioactive sites of Spirulina proteins. Moreover, pepsin hydrolysate, being highly hydrophobic, has enhanced ability to inhibit intestinal lipid absorption. Similarly, aromatic amino acid rich-chymotrypsin hydrolysates show improved modulation of pathways involved in lipid metabolism [38]. However, the findings of the current study show that a combination of these hydrolysates, i.e., PTC-SPPH have the most pronounced lipid modulatory effect. Moreover, the trypsin-based hydrolysates showed comparatively greater decline in serum triglycerides, emphasizing the critical role of enzyme-specific peptide and its potential variation as compared to other hydrolysates.

Since dyslipidemia and hepatic dysfunction are closely associated with oxidative stress, antioxidant biomarkers were subsequently assessed to further explore the hepatoprotective potential of Spirulina protein hydrolysates. It is important to note that one enzyme-derived hydrolysate might show stronger activity owing to the cleavage site, degree of hydrolysis, optimal conditions, and primarily, the enzyme involved in cleavage. As observed in a parallel study, different enzymes under different conditions produce peptides with a distinct amino acid sequence, thereby producing structural and functional variations [39]. For example, peptides enriched with hydrophobic amino acids exhibit enhanced antioxidant abilities as compared to other hydrolysates. This specific sequence requires it to be exposed by a specific enzyme, thus producing functional differences. Moreover, the cleavage site also determines the type of amino acid sequence exposed. Similarly, small sized hydrolysates are better absorbed and more bioavailable than larger fragments. Importantly, the degree of hydrolysis must be optimal (moderate/high) for stronger bioactivity as low and too high hydrolysis results in loss of desired bioactivity [40].

The marked modulation of antioxidant biomarkers indicates that the beneficial effects of Spirulina hydrolysates extend beyond lipid metabolism and may involve direct antioxidant mechanisms mediated by bioactive peptides. For both serum and liver homogenate, all the biomarkers of oxidative stress, including MDA, SOD, CAT, and GSH, showed statistically significant differences across all study groups as indicated by *p* < 0.05. Regarding serum MDA levels, the peak reduction was noted in the treatment group PTC-SPH (88.67%, 0.024 U/L), indicating the maximum protective effect of Spirulina hydrolysates, followed by SPPE (77.35%, 0.048 U/L).

In terms of serum SOD levels, maximum increase was observed for PTC-SPH (312.65%, 77.134 U/L), followed by Positive Control (295.49%, 73.925 U/L), while the least increase was noted in chymotrypsin-SPPH (72.66%, 32.274 U/L) as compared to the Negative Control (18.692 U/L). As per serum CAT concentration, the highest level was observed for the Positive Control group (145.71%, 18.023 U/L), while the minimum level was depicted in pepsin-SPH (41.03%, 10.345 U/L) as compared to the Negative Control (7.335 U/L). For serum GSH levels, chymotrypsin-SPPH showed a remarkable increase (370.96%, 0.292 U/L), followed by PTC-SPPH (279.03%, 0.235 U/L); whereas, trypsin-SPPH showed a minimum increase (141.93%, 0.15 U/L) as compared to Negative Control (0.062 U/L). Regarding liver homogenate, MDA levels were reduced remarkably in chymotrypsin-SPPH group (73.07%, 0.41 U/L), indicating the highest protective effect, while the least decrease was observed in the PTC-SPPH group (46.74%) in comparison with the Negative Control (1.523 U/L).

As per SOD levels, maximum increment was depicted by PTC-SPPH (588.97%, 26.691 U/L), indicating the highest antioxidant potential, followed by trypsin-SPPH group (308.38%, 15.821 U/L) as compared to the Negative Control (3.874 U/L). In terms of increase in CAT levels, trypsin-SPH showed the highest increase (24.22%, 16.728 U/L), indicating an improved antioxidant status, followed closely by the Positive Control group (23.98%, 16.696 U/L), while the minimum increase was noted for SPEE treatment group (17.31%, 15.798 U/L) in comparison with the Negative Control (13.466 U/L). For an increment in GSH levels, the treatment group PTC-SPPH marked the peak levels (406.94%, 2.19 U/L); whereas, least level was indicated by treatment group pepsin-SPPH (25.23%, 0.541 U/L) as compared to the Negative Control (0.432 U/L) (Figure 2).

These peptide-dependent antioxidant properties are consistent with the improved redox balance observed in both serum and hepatic tissues in the present study. Bioactive peptides, specific protein fragments having 2–30 amino acids, possess certain bioactive potential and ultimately confer health benefits. These bioactive peptides are derived from proteins via enzymatic hydrolysis to achieve their activity and have a unique sequence of amino acids. The bioactivity of these peptides depends primarily on this distinct amino acid sequence revealed by specific hydrolytic enzymes [40]. For example, peptides with abundant hydrophobic and aromatic amino acids may interact more precisely with the plasma membranes and thus play radical scavenging role against lipid peroxidation. Similarly, positively charged amino acids play their antioxidant role via metal chelation thereby preventing the catalysis of lipid peroxidation process by these metals. Notably, molecular weight/size of these peptides, as determined by the composition, also determines the bioavailability and ultimately bioactivity of these bioactive peptides. For instance, shorter chain peptides possess stronger bioactive abilities as compared to larger peptides [41].

The protective role of these hydrolysates against oxidative stress can further be understood by examining the physiological significance of individual antioxidant biomarkers involved in cellular defense systems. The improved antioxidant status in both the serum and the liver tissue, as indicated by the results of the current study, is one of the core mechanisms that account for the hepatoprotective abilities of Spirulina protein hydrolysates. As metabolic complications and liver diseases result from oxidative stress, Spirulina hydrolysates have been found to significantly increase the activity of endogenous antioxidant enzymes, such as superoxide dismutase (SOD), catalase (CAT), glutathione peroxidase (GPx), and glutathione reductase in hepatic tissue. Moreover, a parallel increase in non-enzymatic antioxidants, such as reduced glutathione (GSH) and total antioxidant capacity in serum is also observed. These observations suggest that bioactive peptides can perform antioxidant role by modulating intracellular redox signaling pathways. Furthermore, the observed decrease in lipid peroxidation markers such as malondialdehyde (MDA) and thiobarbituric acid-reactive substances (TBARS) in liver tissue supports the actual effect of Spirulina hydrolysates in scavenging free radicals before they are able to damage cellular membranes, proteins and DNA.

As supported by a parallel study, this effective antioxidant defense system in the liver tissues protects the liver from acute as well as chronic damage associated with multiple factors such as aging, toxins exposure and metabolic stress [34]. Thus, the correlation of increased serum antioxidant markers to improved antioxidant enzymes activity in the liver suggests that the Spirulina protein hydrolysates offer a network of protection in different pathological conditions associated with oxidative stress.

Since oxidative stress and lipid dysregulation ultimately contribute to hepatocellular injury, liver function biomarkers were further analyzed to determine whether these biochemical improvements translated into functional hepatic protection. The effectiveness of Spirulina hydrolysates as hepatoprotective agents can be well interpreted in terms of role of antioxidant biomarkers like SOD, CAT, GPx, GSH, MDA and TAC in reducing oxidative stress level in biological systems. For instance, Superoxide dismutase (SOD), a part of the enzymatic defense mechanism, is the enzyme responsible for converting superoxide radicals into hydrogen peroxide and oxygen to prevent cellular membranes from ROS [42]. In the present study, elevated levels of SOD after administration of Spirulina suggests increased ability to neutralize superoxide anions generated in normal as well as pathological conditions. Synergistically, Catalase (CAT) and glutathione peroxidase (GPx) are responsible for removing hydrogen peroxide from the cell since CAT operates mainly in the peroxisomes and GPx in the cytosol and mitochondria using reduced glutathione as a cofactor. Importantly, the ratio of reduced glutathione (GSH) to oxidized glutathione (GSSG), a sensitive indicator of redox balance in hepatocytes, shows that an elevated GSH concentration and GSH/GSSG ratio indicate a cellular environment able to resist oxidative damage [43].

Additionally, lipid peroxidation markers, such as Malondialdehyde (MDA) indicate oxidative damage to cellular membranes. In the present study, reduced MDA levels after administration of Spirulina protein hydrolysates indicate decreased oxidative damage and increased liver tissue integrity [44]. Moreover, total antioxidant capacity (TAC) is an integrated measure of total antioxidant compounds in biological samples, which includes both the enzymatic and non-enzymatic antioxidants (vitamins C and E, polyphenols, and other free-radical-scavengers). In the present study, increased TAC values suggest higher antioxidant species against oxidative stress. Therefore, the overall modulation of these parameters provides robust evidence for the hepatoprotective potential of Spirulina protein hydrolysates, enabling more detailed exploration of the pathways involved in the antioxidant activity.

The observed bioactivity of these Spirulina hydrolysates can be partly explained by our previous study, which was performed to assess the nutritional profiling and antioxidant potential of these Spirulina hydrolysates [23]. These hydrolysates predominantly consisted of low molecular weight peptides (<10 kDa), along with increased antioxidant abilities (DDPH and FRAP) and reduced lipid peroxidation. Moreover, these hydrolysates also contained a higher number of phenolics, flavonoids and terpenoids than whole Spirulina protein extract. These characteristics may have improved the bioavailability and biological activity of the released peptides, thereby contributing to the observed improvements in lipid metabolism and oxidative stress in the high-fat diet-fed rats.

The observed reductions in liver enzymes and bilirubin levels suggest improved hepatic integrity, warranting further interpretation in the context of hepatoprotective mechanisms of Spirulina-derived peptides. For liver homogenate samples, a statistically significant difference was noted for total bilirubin, liver enzymes including ALT, AST, and AP across all the study groups as inferred by *p* < 0.05. However, the difference in the level of direct and indirect bilirubin was statistically insignificant (*p* > 0.05). The highest reduction in total bilirubin concentration was observed in the treatment group, pepsin-SPPH and Positive Control (92.91% and 92.36%, respectively), while the least decrease was noted in trypsin-SPPH (34.79%) as compared to the Negative Control.

As per direct bilirubin levels, the maximum decrease was shown by the Positive Control (79.06%), while the minimum reduction was observed in chymotrypsin-SPPH (2.30%) as compared to the Negative Control. In terms of indirect bilirubin concentration, a trend similar to total bilirubin concentration was noted. The treatment group pepsin-SPPH and Positive Control showed maximum decrease (94.97% and 93.20%, respectively), followed by treatment groups PTC-SPPH and chymotrypsin-SPPH (90.69% and 90.36%, respectively) in comparison with the Negative Control. Similarly, for liver enzyme ALT, in comparison to the negative control, the peak reduction was noted in the treatment group chymotrypsin-SPPH (70.94%), followed by PTC-SPPH (68.91%).

Interestingly, AST and AP followed an identical pattern, with the highest reduction observed in the Positive Control (76.79% and 72.03% for AST and AP, respectively) and the lowest reduction in pepsin-SPPH (28.62% and 27.91% for AST and AP, respectively). Regarding serum samples, a statistically significant difference was noted for total bilirubin, indirect bilirubin, and liver enzymes, including ALT, AST, and AP, across all the study groups as inferred by *p* < 0.05. Nevertheless, the difference in the level of direct bilirubin was statistically insignificant (*p* > 0.05). For serum total bilirubin and indirect bilirubin, an identical trend was observed, with the Positive Control showing the highest decline (88.63% and 92.84%, respectively), followed closely by the treatment group chymotrypsin-SPPH (87.68% and 90.19%, respectively) as compared to the Negative Control (Table 2).

In terms of serum ALT, AST, and AP levels, all the study groups showed comparable reduction compared to the Negative Control. For ALT levels, chymotrypsin-SPPH showed the highest decline (67.56%). Moreover, maximum decrease in serum AST was noted in treatment group pepsin-SPPH (6.58%), and for AP reduction, peak decline was observed for trypsin-SPPH (6.21%), as compared to the Negative Control. In terms of hepatoprotective potential, the tested intervention can be well interpreted by analyzing the hepatic function markers. In the present study, a significant modulation of liver enzymes such as alanine aminotransferase (ALT), aspartate aminotransferase (AST), and alkaline phosphatase (ALP) resulted from the Spirulina protein hydrolysates. As excursions in the level of these enzymes indicate underlying pathologies such as liver injury due to hyperlipidemia or oxidative stress, thus they can be used as biomarkers. Thus, the reduction in the levels of these liver enzymes can be attributed to the protective action of Spirulina protein hydrolysates on cell membranes [45].

As these bioactive peptides serve their anti-inflammatory roles and reduce oxidative stress in liver cells, thereby preventing cell destruction and release of liver enzymes into blood. This effect was well exhibited in the outcomes of the current study as an overall hepatic and biliary function improvement related to marked reduction in ALP levels. Overall, it can be suggested that Spirulina protein hydrolysates may serve as potent nutraceuticals for metabolic disorders such as dyslipidemia and hyperlipemia. To further validate the biochemical findings, microscopic examination of hepatic tissues was performed to assess structural and morphological alterations across the treatment groups.

**Figure 3:**
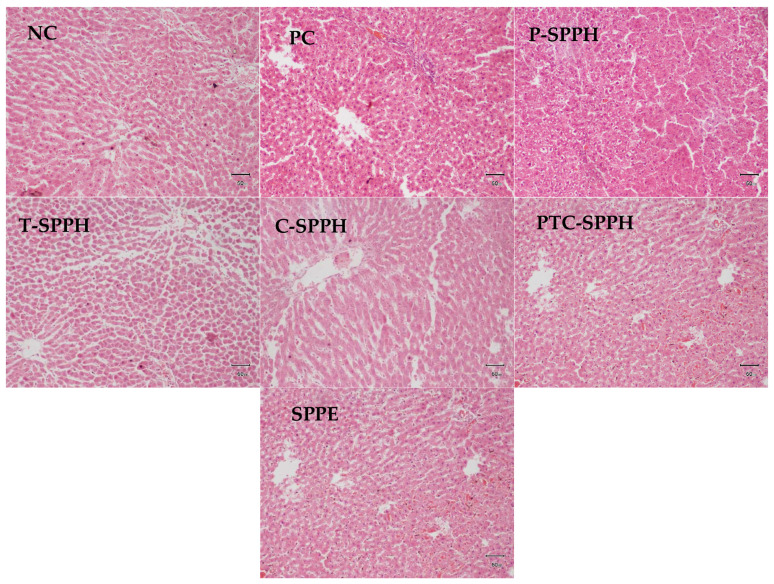
Impact of the administration of Spirulina protein-based hydrolysate on liver histopathology in HFD-induced hyperlipidemia rat model. NC: Negative Control; PC: Positive Control; SPPH: Spirulina platensis protein hydrolysates; P-SPPH: Pepsin-SPPH; T-SPPH: Trypsin-SPPH; C-SPPH: Chymotrypsin-SPPH; PTC-SPPH: Pepsin-Trypsin-Chymotrypsin-SPPH; Image-JPEG, Scale Bar-50 mm, H/E staining, Magnification-10X, Microscope CX-33-Olympus, United Kingdom, Olympus Imaging Software, Version DP-20.

Microscopic evaluation of hepatic tissues across all groups revealed varying degrees of morphological alterations. The Negative Control exhibited mild to moderate microvascular fatty changes with preserved vascular structures and no evidence of inflammation, necrosis, or fibrosis. The Spirulina protein extract group demonstrated mild to moderate hydropic degeneration predominantly in the centrilobular hepatocytes, accompanied by sinusoidal congestion. The PTC (pepsin–trypsin–chymotrypsin) hydrolysate group showed mild fatty changes confined mainly to the centrilobular area, with intact midzonal hepatocytes and no alterations in sinusoidal, central venous, or portal structures. Trypsin hydrolysates presented normal hepatic architecture, with well-organized hepatic cords and an unremarkable central vein, sinusoids, and portal area, reflecting features of healthy liver tissue. Chymotrypsin hydrolysates similarly displayed mild centrilobular fatty change without structural disruption in other hepatic regions. In contrast, pepsin hydrolysates induced moderate to marked cellular swelling in the centrilobular hepatocytes, extending mildly into the midzonal area. The Positive Control showed moderate hydropic degeneration in centrilobular hepatocytes with intact vascular structures but mild portal infiltration. Collectively, these findings indicate variable hepatic responses depending on the type of Spirulina-derived preparation, with some hydrolysates demonstrating protective or minimal alterations, while others produce mild to moderate degenerative changes. Overall, the histopathological observations corroborate the biochemical and antioxidant findings, collectively supporting the hepatoprotective and metabolic regulatory potential of Spirulina protein hydrolysates (Figure 3). The heterogenicity in the biological effectives of these hydrolysates was mainly due to their unique enzyme cleavage pattern leading to generation of exclusive peptides with diverse biological activity and potency. Because the produced hydrolysates with peptides can vary on the basis of composition of amino acids, molecular weight (particularly smaller ones producing potent bioactivity), and the property of hydrophobicity [46]. These conformational differences may influence their absorption and ability to interact with metabolic pathways resulting in desired bioactivity. These hydrolysates may be attributed to alterations in lipid metabolic pathway metabolites involving downregulation of lipogenic regulators, i.e., SREBP-1c, ACC and PPAR γ and upregulation of lipid oxidation pathways, mainly AMPK and PPAR α [47]. Nonetheless, the present study was limited to biochemical testing and antioxidant assays, the inclusion of genetic biomarkers and identification of specific peptide sequences generated in these hydrolysates should also be done to study its impact on the expression on players involved in lipid metabolism and oxidative stress can be done to further validate these results.

Another limitation of the study is the absence of a normal control group, which would have provided a comparative insight into a physiological baseline for biochemical parameters. However, our study was primarily focused on assessing the relative efficacy of Spirulina hydrolysates in HFD fed rat model. Future studies can add a normal control group to validate baseline biochemical parameters. Our study was also limited to small sample size with only six animals per group. As no formal priori power calculation was performed. However, study similar to ours commonly used 5-8 animals which are sufficient to detect medium to large effects (Cohen’s d ≈ 1) with estimated power of 70–80% (α = 5%). A larger sample size perhaps would have increased statistical robustness.

## 4. Conclusions

Overall, Spirulina-derived protein hydrolysates exhibited strong therapeutic potential across multiple physiological domains. The treatment groups consistently showed improvements in obesity-related anthropometric markers, with notable reductions in BMI and Lee Index, particularly in the Positive Control and PTC-SPPH groups, indicating effective weight-modulating properties. Although increments in abdominal and thoracic circumferences varied among groups, the overall decline in the AC: TC ratio in most treatments suggests a favorable shift toward healthier body fat distribution.

Protein metabolism markers demonstrated that Spirulina hydrolysates enhanced hepatic and serum protein status, as evidenced by significant increases in albumin and total protein concentrations, with the Positive Control showing the strongest responses repeatedly. Improvements in lipid metabolism were evident in both serum and liver homogenate, where all treatments produced significant reductions in total cholesterol, triglycerides, and LDL. PTC-SPPH and Positive Control were particularly effective, while HDL changes remained statistically insignificant despite favorable trends in select groups. Liver function markers also reflected a protective and restorative effect, with substantial declines in bilirubin levels and liver enzymes, especially in the Positive Control, Chymotrypsin-SPPH, and PTC-SPPH groups. Similarly, renal function showed partial improvement, with significant reductions in uric acid and BUN, highlighting potential nephroprotective effects, mainly in Positive Control and enzyme-specific hydrolysates.

Finally, all treatment groups demonstrated robust antioxidant responses in both serum and liver homogenate. Marked reductions in MDA and substantial increases in SOD, CAT, and GSH confirmed enhanced oxidative stress resistance, with PTC-SPPH consistently emerging as one of the strongest performers. Collectively, these findings indicate that Spirulina protein hydrolysates, particularly PTC-SPPH, Chymotrypsin-SPPH, Trypsin-SPPH, and the Positive Control formulation, exert comprehensive beneficial effects on metabolic health, liver and renal function, and oxidative balance. This supports their potential application as functional bioactive agents for managing obesity-related complications and improving overall physiological homeostasis.

## Figures and Tables

**Table 1:** Impact of Spirulina protein hydrolysates administration on protein profile in HFD-induced hyperlipidemic rat model.

Groups	Liver Homogenate (mg/mL)		Serum (mg/dL)	
	Albumin	Total Proteins	Albumin	Total Proteins
Negative Control	0.38 c ± 0.03	2.83 ± 0.13	2.21 c ± 0.07	14.3 ± 0.41
Positive Control	0.87 a ± 0.31	7.76 ± 5.08	2.87 a ± 0.29	17.47 ± 2.1
SPPE	0.65 abc ± 0.04	5.23 ± 0.87	2.34 bc ± 0.09	16.14 ± 0.81
Pepsin-SPPH	0.54 bc ± 0.05	6.42 ± 0.71	2.51 bc ± 0.11	16.75 ± 2.42
Trypsin-SPPH	0.65 abc ± 0.06	4.99 ± 0.53	2.41 bc ± 0.1	16.32 ± 0.25
Chymotrypsin-SPPH	0.73 ab ± 0.06	7.26 ± 0.68	2.34 bc ± 0.17	17.53 ± 1.51
PTC-SPPH	0.6 abc ± 0.06	4.21 ± 0.11	2.58 b ± 0.04	15.41 ± 1.46
*p* value	0.029 *	0.200	0.001 *	0.101

Data presented as mean ± SD; one-way analysis of variance (ANOVA) applied. Intergroup differences were evaluated using Duncan Multiple Range Test (DMRt). Heterogenous letters in a column represent significant different groups. The level of significance was kept at 5%, * significant difference.

**Table 2:** Impact of Spirulina protein hydrolysates administration on liver enzymes in HFD-induced hyperlipidemic rat model.

Groups	Liver Homogenate (mg/mL)					
	Total Bil	Direct Bil	Indirect Bil	ALT (U/mL)	AST (U/mL)	AP (U/mL)
Negative Control	2.4 a ± 0.78	0.14 ± 0.12	2.26 a ± 0.9	10.59 a ± 2.83	7.42 a ± 0.91	8.37 a ± 0.85
Positive Control	0.18 b ± 0.12	0.03 ± 0.02	0.15 b ± 0.14	5.02 bc ± 0.34	1.72 d ± 0.59	2.34 c ± 0.9
SPPE	0.3 b ± 0.25	0.07 ± 0.06	0.24 b ± 0.19	6.05 b ± 0.76	3.96 bc ± 2.2	4.62 bc ± 2.24
Pepsin-SPPH	0.17 b ± 0.03	0.06 ± 0.05	0.11 b ± 0.06	5.47 bc ± 0.08	5.29 b ± 0.49	6.03 ab ± 2.43
Trypsin-SPPH	1.57 ab ± 1.85	0.08 ± 0.02	1.49 ab ± 1.82	4.31 bc ± 0.15	3.08 cd ± 0.46	4.11 bc ± 2.95
Chymotrypsin-SPPH	0.36 b ± 0.38	0.14 ± 0.05	0.22 b ± 0.35	3.08 c ± 0.88	2.22 cd ± 0.74	3.22 bc ± 0.4
PTC-SPPH	0.27 b ± 0.03	0.06 ± 0.03	0.21 b ± 0.01	3.29 bc ± 0.76	1.82 d ± 0.63	4.14 bc ± 0.01
*p* value	0.013 *	0.295	0.019 *	0.001 *	0.000 *	0.016 *
	**Serum (mg/dL)**					
**Groups**	**Total Bil**	**Direct Bil**	**Indirect Bil**	**ALT (U/L)**	**AST (U/L)**	**AP (U/L)**
Negative Control	1.29 a ± 0.17	0.14 ± 0.13	1.15 a ± 0.27	284.9 a ± 61.93	160.57 a ± 6.37	214.47 a ± 7.16
Positive Control	0.15 b ± 0.13	0.06 ± 0.07	0.08 b ± 0.15	99.13 b ± 6.3	152.58 b ± 2.48	202.93 b ± 2.48
SPPE	0.26 b ± 0.17	0.1 ± 0.15	0.15 b ± 0.15	99.47 b ± 5.58	153.1 b ± 1.64	203.45 b ± 2.7
Pepsin-SPPH	0.27 b ± 0.31	0.03 ± 0	0.24 b ± 0.32	102.4 b ± 7.31	150 b ± 0.5	204.83 b ± 0.8
Trypsin-SPPH	0.26 b ± 0.14	0.05 ± 0.03	0.2 b ± 0.16	99.99 b ± 12.12	151.38 b ± 1.82	201.15 b ± 1.43
Chymotrypsin-SPPH	0.16 b ± 0.17	0.05 ± 0.04	0.11 b ± 0.14	92.41 b ± 5.43	152.76 b ± 1.78	201.9 b ± 1.03
PTC-SPPH	0.41 b ± 0.46	0.11 ± 0.09	0.3 b ± 0.38	122.27 b ± 18.27	151.15 b ± 1.05	203.22 b ± 3.18
*p* value	0.000 *	0.783	0.000 *	0.000 *	0.008 *	0.002 *

Data presented as mean ± SD; one-way analysis of variance (ANOVA) applied. Intergroup differences were evaluated using Duncan Multiple Range Test (DMRt). Heterogenous letters in a column represent significant different groups, Level of significance was kept at 5%, * Significant difference.

## Data Availability

The raw data supporting the conclusions of this article will be made available by the authors upon request.

## Abbreviations

The following abbreviations are used in this manuscript:

SPPH	Spirulina-derived protein hydrolysates
SPPE	*Spirulina platensis* protein extract
BMI	Body Mass Index
LDL	Low Density Lipoprotein
HDL	High Density Lipoprotein
ALT	Alanine transaminase
AST	Aspartate aminotransferase
BUN	Blood Urea Nitrogen
MDA	Malondialdehyde
SOD	Superoxide dismutase
CAT	Catalase
GSH	Glutathione
HFD	High-Fat Diet
PBS	Phosphate-Buffered Saline
TGs	Triglycerides
AP	Alkaline phosphatase
TBARS	Thiobarbituric Acid-Reactive Substances
TCA	Trichloroacetic acid
TBA	Thio barbituric acid
NBT	Nitro Blue Tetrazolium
PMS	Phenazine Methosulfate System
DTNB	5,5-dithiobis (2-nitrobenzoic acid)
